# DP2: Distributed 3D image segmentation using micro-labor workforce

**DOI:** 10.1093/bioinformatics/btt154

**Published:** 2013-04-10

**Authors:** Richard J. Giuly, Keun-Young Kim, Mark H. Ellisman

**Affiliations:** National Center for Microscopy and Imaging Research, Center for Research in Biological Systems, Department of Neurosciences, University of California, San Diego, La Jolla, CA 92093, USA

## Abstract

**Summary:** This application note describes a new scalable semi-automatic approach, the Dual Point Decision Process, for segmentation of 3D structures contained in 3D microscopy. The segmentation problem is distributed to many individual workers such that each receives only simple questions regarding whether two points in an image are placed on the same object. A large pool of micro-labor workers available through Amazon’s Mechanical Turk system provides the labor in a scalable manner.

**Availability and implementation:** Python-based code for non-commercial use and test data are available in the source archive at https://sites.google.com/site/imagecrowdseg/.

**Contact:** rgiuly@ucsd.edu

**Supplementary information:** Supplementary data are available at *Bioinformatics* online.

## 1 INTRODUCTION

The improved resolution and amount of detail afforded by emerging electron microscopy (EM) techniques, such as serial block scanning EM (SBEM) (Denk and Horstmann, 2004; Leighton, 1981), is enabling researchers to explore scientific questions pertaining to morphology and network connectivity that were previously impossible (Eisenstein, 2009). SBEM techniques, coupled to new staining protocols (Deerinck *et al.*, 2010), are able to reveal cell boundaries, sites such as synapses, and many intracellular components, such as synaptic vesicles and mitochondria.

Manual segmentation represents a well-recognized bottleneck in cellular imaging. In a typical scenario, segmentation involves a single trained expert using automated algorithms or manual methods to examine and mark up each individual slice and trace contours around the structures of interest using a program such as TrakEM2 (Cardona, 2006) or other specialized software programs. Dual Point Decision Process (DP2) streamlines and parallelizes the process by distributing it to a large number of workers. Amazon’s Mechanical Turk system enables rapid completion of jobs as a consequence of the large number of workers continuously available and attracted to this resource (In our tests, tens of thousands of decisions were accomplished in <1 day.). Average cost was 1.2 dollars per cubic micron for Dataset 1, a mouse optic nerve sample, and 56 dollars per cubic micron for Dataset 2, a mouse cerebellar neuropil sample (shown in Supplementary Information). The tests were performed with a payment of one cent (US Dollars) per decision.

## 2 BACKGROUND

Although progress has been made to develop automatic segmentation techniques appropriate for cells (Andres *et al.*, 2008; Jain *et al.*, 2007; Jurrus, 2010; Kaynig *et al.*, 2010; Mishchenko, 2009), there remains a need for more accurate, rapid and robust techniques to delineate cells in SBEM data. Jeong and Chklovskii use a combination of 3D visualization and semi-automatic segmentation to address the segmentation challenge (Chklovskii *et al.*, 2010; Jeong *et al.*, 2009). Our method differs from these in that we use micro-labor workers with a simple web interface rather than a trained user with a more complex interface. This makes our method scalable in that a large number of workers are readily available for a given job.

Roberts describes a method using sparse scribbles from users (Roberts *et al.*, 2011) to semi-automatically trace neural processes. Additionally, the Eyewire project led by Sebastian Seung uses volunteers who learn to perform 3D semi-automatic segmentation with a specialized web viewer (Wiecek, 2012). Our method differs from these in that user decisions are completely binary based on images of points within superpixels. Additionally, our application uses the micro-labor environment of Amazon’s Mechanical Turk so that work can be accomplished practically by the existing pool of visitors who survey Mechanical Turk offerings. To make our process appropriate for such a system, users are given a simple presentation of dots automatically rendered on an image and only asked to answer yes or no decisions. Also, in contrast to Eyewire, we test with membranes and intracellular components stained, whereas Eyewire currently addresses data where only extracellular regions are stained. Finally, although some other methods such as (Jain *et al.*, 2007) use machine learning for automatic segmentation, our method does not; therefore, we do not need a large amount of human-labeled training data or a large of amount of training time for machine learning.

## 3 METHODS

The overall DP2 process consists of an initial over-segmentation followed by a manual merging process to create a segmentation. The 3D dataset is represented as a stack of 2D images.

To pre-process the data, we first smooth the original images with a 2D Gaussian filter. Then a 2D watershed operation from the Insight Toolkit (Yoo *et al.*, 2002) is applied on the gradient magnitude of the Gaussian-filtered image to create superpixels. Each region of the watershed operation is a superpixel, and the watershed operation is tuned to favor over-segmentation rather than under-segmentation. This superpixelization process is applied to every slice of the 3D volume to produce an initial over-segmentation.

To begin the merge decision process, a graph is created to represent the superpixels and possible merge operations. Each of the superpixels is a node of the graph, and each pair of adjacent superpixels represents a possible edge in the graph. Any two neighboring superpixels in a 2D image are considered adjacent (Case 1). Additionally, any two superpixels in neighboring planes that touch are considered adjacent (Case 2). The goal of the workers is to decide what adjacent superpixels should be merged to transform the over-segmentation into a more accurate segmentation. Therefore, if users judge that adjacent superpixels should be connected, the corresponding edge in the graph is added. Two positive votes from independent users are required for the merge to be accepted. After graph edges are added, the final step consists of computing connected components in the graph. Each component represents a single object of the image, such as an axon belonging to a nerve cell.

**Fig. 1.**
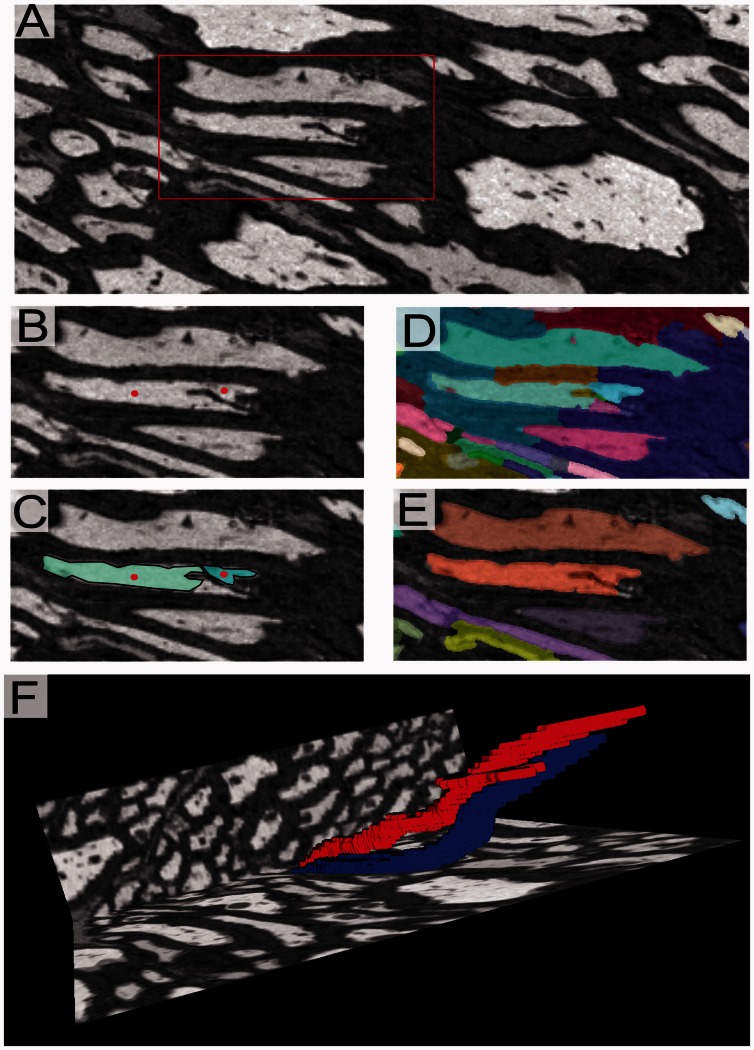
(**A**) Image of axons and myelin in optic nerve. (**B**) Red dots placed on two different superpixels. (**C**) Two superpixels associated with two dots presented to the user. (**D**) All superpixels in the view, highlighted in different colors. (**E**) Segmentation after all superpixels merge decisions have been made. (**F**) 3D rendering showing two axons in red and blue

Careful presentation of decisions to the workers in the merging decision process is important. To keep presentation clear and elegant, they are not shown the outline of superpixels explicitly. Rather, for each pair of adjacent superpixels, an image is generated (as shown in Fig. 1B) that shows two dots superimposed on the original image data placed at the two superpixel centers (If the center of a superpixel lies outside of the superpixel, a random point inside of the superpixel region is used instead.). The images are cropped so that the field of view only covers the two superpixels relevant to the decision. For each merge decision, the job of the user is to decide whether the two dots lie on the same object. If both dots lie in the same object, then the superpixels should be merged.

The implementation of the merging process is demonstrated using Mechanical Turk. Two types of tasks are assigned to users. One is to merge two adjacent superpixels in one image plane (Case 1), and the other is to connect superpixels in adjacent planes (Case 2). For Case 1, the user makes merge decisions based on whether two dots on a single image corresponding to two adjacent superpixels are within the same cell. For Case 2, the worker must identify superpixels that should be connected from one image slice to the next. To accomplish this, a two-frame animated image is presented that repetitively switches between the two image slices, showing a dot on each the two superpixels that are being evaluated. In this case, the user must judge whether the dot is staying inside the same cell as frames alternate.

## Supplementary Material

Supplementary Data
