# Supplementary material for: DP2: Distributed 3D image segmentation using micro-labor workforce
Source: Bioinformatics. 2013 Apr 10;29(10):1359–60. doi: 10.1093/bioinformatics/btt154 (PMC3654713; doi:10.1093/bioinformatics/btt154)
Supplement: Supplementary Data [file supp_29_10_1359__index.html]

DP2: Distributed 3D Image Segmentation Using Micro-labor Workforce — DP2: Distributed 3D image segmentation using micro-labor workforce — DP2: Distributed 3D image segmentation using micro-labor workforce — Supplementary Data 

# DP2: Distributed 3D image segmentation using micro-labor workforce

## Supplementary Data

files

**Files in this Data Supplement:**

- Supplementary Data - doc file
