# Supplementary material for: DP2: Distributed 3D image segmentation using micro-labor workforce
Source: Bioinformatics. 2013 Apr 10;29(10):1359–60. doi: 10.1093/bioinformatics/btt154 (PMC3654713; doi:10.1093/bioinformatics/btt154)
Supplement: Supplementary Data [file supp_btt154_supplementary_text2.doc]

**
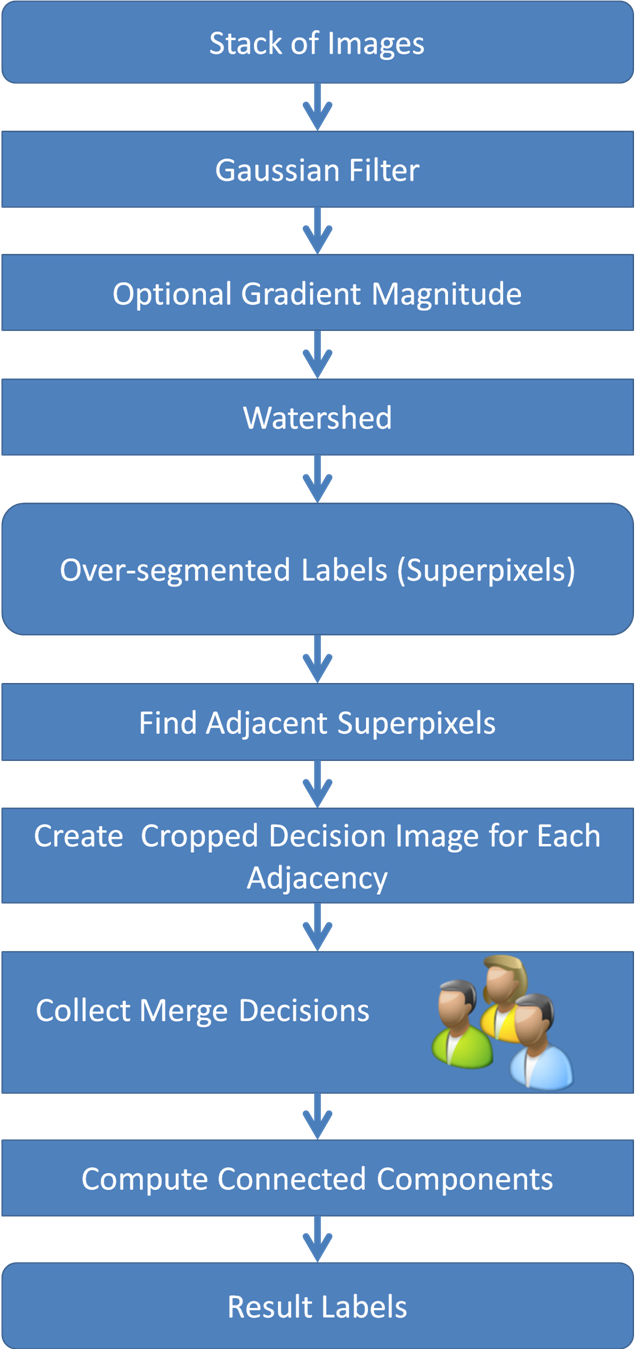
TEST RESULTS**

Fig. 2. DP2 Process Flowchart.

Two SBEM datasets were used for testing. Dataset 1 contains mouse optic nerve axons and has dimensions 14 microns × 14 microns × 5 microns. It was acquired using a 9.4 nm × 9.4 nm × 70 nm voxel size. Dataset 2 contains more complex image data, mouse cerebellar neuropil with unmylinated processes, and has dimensions 3.5 microns × 3.5 microns × 0.25 microns. It was acquired using 10nm × 10nm × 50nm voxel size.

Our first test was performed on Dataset 1, a 3D image of the mouse optic nerve (see Figure 1A). Figure 1D shows the over-segmentation of an image using the watershed method described previously. Parameters for the watershed operation were threshold=0.00015 and level=0.35 (Ibane*z et a*l., 2003). Note the example of an over-segmented region shown in Figure 1C. This over-segmentation was corrected by a decision from Mechanical Turk. A user was presented with an image as in Figure 1B and made the decision that both dots were inside the same cell process, which implies that the two superpixels in Figure 1C should be merged. Figure 1E shows the result after users have merged superpixels shown in Figure 1D. For Dataset 1, all objects of thickness in Z of 2 voxels or less were assumed to be errors and not included in the results. Approximately 85 different axons were segmented in the dataset. To accomplish this, a total of 84,849 decisions were requested from users. Thirty six percent of the decisions were in-plane (Case 1), and 64% were for neighboring planes (Case 2). Figure 1F shows a 3D rendering of two axons. Other axons are hidden to make the rendering less cluttered.

Our second test was performed on Dataset 2, a 3D image of mouse cerebellum tissue (see Figure 3A). Mitochondria and cells are were segmented as separate objects for this test. Parameters for the watershed operation were threshold=0.00015 and level=0.30. Processing differed from processing of Dataset 1 in the following ways. First, to accommodate the thinner dark borders between cells, the watershed operation was performed directly on the Gaussian filtered image. (The gradient magnitude was not used.) Second, the points presented to the user were the maximal (most white) pixels on a given superpixel in the Gaussian filtered image. This point selection method helped to ensure that the displayed points were not on a dark border between cells, which can confuse the decision process. Figure 3B shows the result after users have merged superpixels. A total of 18,483 decisions were requested from users. Forty three percent of the decisions were in-plane (Case 1), and 57% were for neighboring planes (Case 2).

Using DP2, two possible sources of error are (1) under-segmentation of the data at the watershed operation and (2) incorrect merge choices from users. Under-segmentation errors result in superpixels that extend across the border of a cell. These errors are not corrected with the current process, but they are rare because the watershed segmentation tends to over-segment rather than under-segment. For Dataset 1, a total of 19,588 superpixels were detected, and out of these, 23 under-segmentation errors occurred during the watershed process. To estimate accuracy of human merge decisions, 600 decisions were selected at random and checked by an expert user. Out of these, 2.8% of individual user decisions were found to be errors. For Dataset 2, no under-segmentation of cells occurred during the watershed process. To estimate accuracy of human merge decisions, 600 decisions were selected at random and checked by an expert user. Out of these, 4.2% of individual user decisions were found to be errors.

The scalability of DP2 stems from the large and growing number of workers who participate with Amazon's Mechanical Turk system. The Mechanical Turk system had 100,000 workers in over 100 countries in 2007 (Jason Pontin, 2007) and over 500,000 workers from over 190 countries in 2011 (Natala, 2011). In our tests, 160 workers contributed to processing Dataset1, and 171 workers contributed to processing Dataset 2. Once the task was configured, collecting results was a convenient process: the workers found the job, trained themselves with given example questions, and then completed the work without need of direct interaction with the employer.

**DISCUSSION**

As shown with our tests, DP2 will have varying performance depending on the complexity of the data. The practical suggested use of DP2 is to accomplish a bulk of segmentation with distributed micro-labor and then allow an expert to proofread the result. In this way, the expert is not burdened with a very large segmentation task. In any case, errors may occur during the DP2 process. For example, the watershed operation can make under-segmentation errors, which will not be corrected by workers. These are minimized by tuning the watershed operation to over-segment, but not always eliminated completely. Also, workers can make over or under-segmentation errors if they select incorrectly. In cases where more exact accuracy is required, we expect that an expert user will proofread and correct DP2 segmentation result with a program such as TrakEM2 in ImageJ, which allows arbitrary corrections to the labels.

For the tests reported here, two independent users were required to vote positively for a merge decision to be accepted. A subject of future work is determining how the number of users per decision affects overall accuracy. Although more user votes per decision may improve the accuracy, requiring more votes per decision will also increase the cost proportionally.

The cost of DP2 varies with the complexity of the data. Highly detailed datasets often require more superpixels, which implies more adjacencies and more decisions. The average cost for Dataset 1 was 1.2 US dollars per cubic micron, and the average cost for Dataset 2 was 56 US dollars per cubic micron. An expert user should choose watershed parameters (threshold and level) that favor over-segmentation without increasing the cost of processing excessively. This is a judgement call of the part of the expert user and the decision depends on the desired accuracy and the amount of expert cleanup the user is willing to perform after DP2 completes.

The purpose of this report is only to demonstrate use of a micro-labor work-force for superpixel merge decisions in stacks of 2D image slices, and therefore a relatively simple method of initial segmentation is used, 2D watershed. We chose to use 2D processing followed by linking from plane to plane, similar to (Chklovski*i et a*l., 2010), rather than fully 3D supervoxelization because resolution in the XY plane for our data is significantly better than effective Z resolution, which is based on microtome step size.

On complex data, such as Dataset 2, cost of processing becomes a more significant factor. In such cases, more advanced initial segmentation methods may be preferred to reduce cost. As a subject of future work, we plan to investigate use of more accurate initial segmentations using more advanced automatic methods such as watershed operation performed on a membrane probability map (Andre*s et a*l., 2008). Use of such initial segmentations may reduce the overall cost by reducing the number of merge decisions.


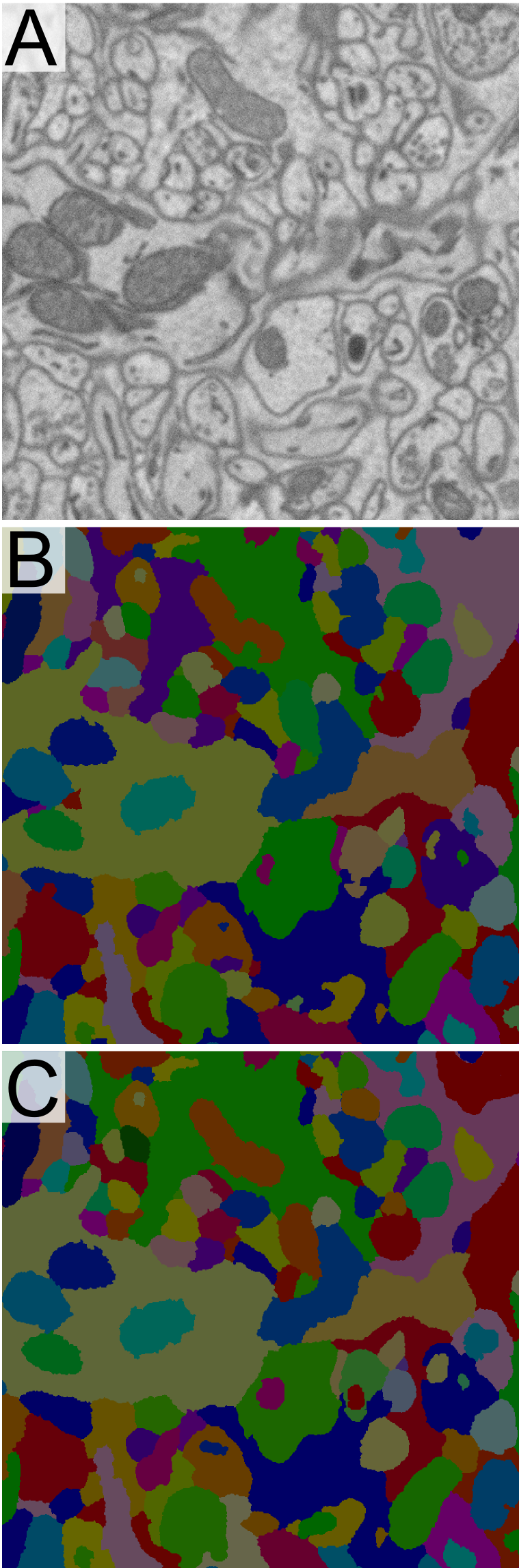


**Fig. 3. Comparison of DP2 result to expert verified labeling for Dataset 2. (A) Image from Dataset 2, mouse cerebellum. (B) Result from DP2 process. (C) Expert labeling.**

**SUPPLIMENTAL REFERENCES**

Andres,B. *et al.* (2008) Segmentation of SBFSEM volume data of neural tissue by hierarchical classification. *Pattern recognition*, 142–152.

Chklovskii,D.B. *et al.* (2010) Semi-automated reconstruction of neural circuits using electron microscopy. *Current opinion in neurobiology*, **20**, 667–675.

Ibanez,L. *et al.* (2003) The ITK software guide.

Jason Pontin (2007) Artificial Intelligence, With Help From the Humans. *The New York Times*.

Natala (2011) AWS Developer Forums: MTurk CENSUS: About how many workers were on Mechanical Turk in 2010?
